# Effect of stocking density of fish on water quality and growth performance of European Carp and leafy vegetables in a low-tech aquaponic system

**DOI:** 10.1371/journal.pone.0217561

**Published:** 2019-05-30

**Authors:** Carmelo Maucieri, Carlo Nicoletto, Giampaolo Zanin, Marco Birolo, Angela Trocino, Paolo Sambo, Maurizio Borin, Gerolamo Xiccato

**Affiliations:** 1 Department of Agronomy Food Natural resources Animal and Environment (DAFNAE), University of Padova, Legnaro, Padova, Italy; 2 Department of Comparative Biomedicine and Food Science (BCA), University of Padova, Legnaro, Padova, Italy; Penn State University, UNITED STATES

## Abstract

Aquaponics (AP) is a semi-closed system of food production that combines aquaculture and hydroponics and represents a new agricultural system integrating producers and consumers. The aim of this study was to test the effect of stocking densities (APL, 2.5 kg m^-3^; APH, 4.6 kg m^-3^) on water quality, growth performance of the European Carp (*Cyprinus carpio* L.), and yield of leafy vegetables (catalogna, lettuce, and Swiss Chard) in a low-technology AP pilot system compared to a hydroponic cultivation. The AP daily consumption of water due to evapotranspiration was not different among treatments with an average value of 8.2 L d^-1^, equal to 1.37% of the total water content of the system. Dissolved oxygen was significantly (p < 0.05) different among treatments with the lowest median value recorded with the highest stocking density of fish (5.6 mg L^-1^) and the highest median value in the hydroponic control (8.7 mg L^-1^). Marketable yield of the vegetables was significantly different among treatments with the highest production in the hydroponic control for catalogna (1.2 kg m^-2^) and in the APL treatment for Swiss Chard (5.3 kg m^-2^). The yield of lettuce did not differ significantly between hydroponic control and APL system (4.0 kg m^-2^ on average). The lowest production of vegetables was obtained in the APH system. The final weight (515 g vs. 413 g for APL and APH, respectively), specific growth rate (0.79% d^-1^ vs. 0.68% d^-1^), and feed conversion (1.55 vs. 1.86) of European Carp decreased when stocking density increased, whereas total yield of biomass was higher in the APH system (4.45 kg m^-3^ vs. 6.88 kg m^-3^). A low mortality (3% on average) was observed in both AP treatments. Overall, the results showed that a low initial stocking density at 2.5 kg m^-3^ improved the production of European Carp and of leafy vegetables by maintaining a better water quality in the tested AP system.

## 1. Introduction

Aquaponics (AP), the integrated multi-trophic production of fish and plants in a semi-closed synergetic recirculating system, is one of the newest sustainable systems of food production [1–4]. In AP systems, the biological wastes excreted by fish (e.g., ammonia, salts) and those generated from the microbial breakdown of feed for fish (i.e., nitrite and nitrate) are absorbed by plants as nutrients for growth. Thus, this method allows the removal of undesirable nutrient wastes from the water by plants and the reuse of the water for fish [5]. Indeed, in AP systems, the majority (> 50%) of the nutrients sustaining the optimal growth of plants is derived from the waste originating from feeding the aquatic organisms [6]. Owing to its integrative character and multiple application (ranging from high- to low-technology systems) scenarios, AP is an atypical and complex production system for food [7]. As reviewed by Goddek et al. [2], AP can be considered a sustainable system of agricultural production, i.e., agricultural practices that do not undermine our future capacity to engage in agriculture and those that reduce the inefficiencies of the production process by designing systems that close cycles of nutrients, which is one of the main aspects of an AP [2]. This technique can play a crucial role in the environmental and socio-economic sustainability of smart cities of the future [8]. Finally, AP has been classified as one of the “ten technologies which could change our lives” by the European Union (EU) Parliament [9].

The essential elements of an AP system are a rearing tank for fish, a component for removing suspended solids, a biofilter, a hydroponic unit, and a storage tank for water [10]. It can be realized almost anywhere with relevant social, economic, and environmental benefits. In fact, considering that this technology does not need soil for cultivation, AP systems can be realized on building roofs or under indoor conditions in neglected industrial buildings. Consequently, AP could represent a new agricultural system integrating producers and consumers, due to the short supply chains and the fresh organic food that it enables [8].

Although AP is based on a simple concept, i.e., the use of waste from fish as nutrients for the production of vegetables, its components can vary with different dimensional ratio forming a complete ecosystem that includes three major groups of organisms: fish, plants, and bacteria [11]. Lennard and Leonard [12] have reported that the hydroponic unit based on film technique was less efficient than the medium-based and floating systems in terms of removal of nutrients from water used for rearing fish and yield of vegetables. A component ratio of ≥ 3 m^3^ of floating hydroponic tank to 1 m^3^ of rearing tank for fish has been suggested by Lam et al. [5] because it showed advantages in improving the production of fish and vegetables, and in removing the wastes of nutrients, total suspended solids (TSS), and in reducing the biochemical oxygen demand (BOD_5_) generated from the culture of fish. Different examples of AP systems based on high technology for an urban context are available (https://urbanfarmers.com). Nevertheless, to our knowledge, little information is available about AP systems based on low technology where the load of fish and consequently the water quality can heavily influence both the health of fish and the yield of vegetables.

In view of this, our study aimed to test the effect of rearing full-scaled European Carp (*Cyprinus carpio* L.) at two stocking densities on the water quality, the growth of fish, and the yield of leafy vegetables, i.e. catalogna (*Cichorium intybus* L. Catalogna group), lettuce (*Lactuca sativa* L.), and Swiss Chard (*Beta vulgaris* L. subsp. *vulgaris*, Cicla Group) in a AP system with low-technology and compare it with a hydroponic cultivation.

## 2. Materials and methods

**Fig 1:**
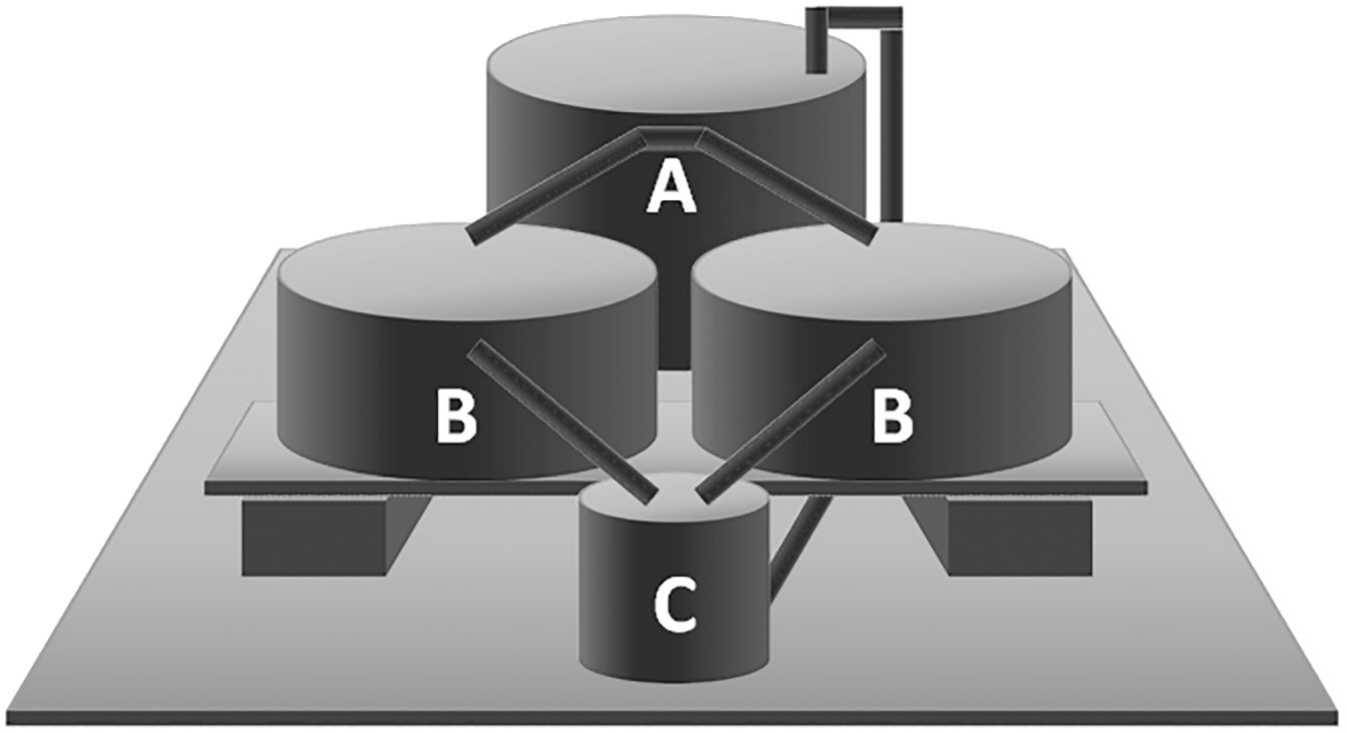
Experimental unit layout. A: tank for fish; B: tank for vegetables/biofilter; C: storage tank for water.

The experimental system was located inside a plastic greenhouse that was 50% shaded at the experimental farm of the University of Padova, North-East Italy (45°20′N, 11°57′E, 6 m a.s.l.). It consisted of nine independent units (Fig 1) divided as follows: three hydroponic units (HP), three AP units with low stocking density of fish (APL), and three AP units with high stocking density (APH). The AP units were designed as “low-technology systems” because they were characterized by: 1) the simplest hydroponic section with the capacity to act as biofilter; 2) the absence of energy to regulate water temperature; 3) the absence of probes for the continuous evaluation of water quality; 4) the absence of probes and systems for remote management; and 5) the absence of devices for water sanitation (UV, ozone).

Each unit consisted of: 1) a main tank (volume 500 L, height 0.80 m) in which the fish were farmed in the AP units or where the nutritive solution was present in the HP units; 2) two tanks for vegetables (volume 275 L each, height 0.35 m), filled with 225 L of expanded clay (LECA Laterlite, Solignano, Italy), that received the same flux of water from the main tank and acted both as biofilter and the hydroponic substrate for vegetable growth; 3) a storage tank for water (volume 50 L, height 0.45 m) where the output from the tanks for vegetables were collected before pumping it back into the main tank (Fig 1). The three parts of the system had water at different heights so that the flow of water was guaranteed by the overflow (from the main tank to the tanks of vegetables, and from the latter ones to the storage tank). A single pump (Newa Jet 1700, NEWA TecnoIndustria Srl, Loreggia, Italy) located in the storage tank returned the water to the main tank. The flow rate was 120 L h^-1^ allowing a complete recirculation of water every 5 hours. The main tanks of the nine units were equipped with a shading cover to prevent fish from jumping and to limit the formation of algae, and a porous stone connected to an aerator (Scubla D100, Scubla Srl, Remanzacco, Italy).

### 2.1 Management of fish and vegetables

The experiment was started on June 19, 2017 and was completed on November 7, 2017. All nine units were filled with the same nutritive solution (220 mg L^-1^ of KH_2_PO_4_, 330 mg L^-1^ of K_2_SO_4_, 456 mg L^-1^ of MgSO_4_·7H_2_O, 31 mg L^-1^ of Fe-EDTA, and 13 mg L^-1^ of micronutrients) at the beginning of the experiment. On June 27, fish were put in the APL and APH units, whereas in the HP units 607 g unit^-1^ of Ca(NO_3_)_2_·4H_2_O was added.

The main tanks were stocked with full-scaled European Carp obtained from a commercial farm (Troticoltura Santa Cristina, Treviso, Italy) with an initial live weight of 169 g ± 56 g at stocking densities of 2.5 kg m^-3^ and 4.6 kg m^-3^ for APL and APH treatments, respectively. The lower stocking density was chosen based on the minimum density capable of providing sufficient N for the growth of plants; the higher stocking density was chosen in view of maintaining the final biomass of fish (estimated as 3–3.5 times the initial weight), below the maximum accepted for organic aquaculture (i.e., 15 kg m^-3^). The fish were manually fed once a day with a commercial compound feed in the form of extruded pellets (Skretting, Verona, Italy; composition: 40% crude protein, 11.5% crude fat, 4% crude fiber, 8% ash, 0.2% sodium, 1.5% calcium, and 0.8% phosphorus, as-fed basis). At the end of the farming period, fish were moved to a commercial aquaculture plant.

During the entire trial, the tanks for vegetables were cultivated in succession with catalogna chicory (*Cichorium intybus* L. Catalogna group, from June 27 to July 25, 9 plants m^-2^), lettuce (*Lactuca sativa* L., from July 26 to August 29, 12 plants m^-2^), and Swiss Chard (*Beta vulgaris* L. subsp. *vulgaris*, Cicla Group, from August 29 to November 7, 10 plants m^-2^), transplanted at the third true leaf stage. Neither pesticides nor antibiotics in water or feed were used during the entire experiment.

### 2.2 Monitoring of water

Throughout the trial, water was not changed in the units. Water lost by evapotranspiration of each unit was manually refilled daily. Two times per week, water was monitored for the following: i) *in situ*, in outflow of the tanks of fish and vegetables: temperature, dissolved oxygen (DO), pH, redox potential (ORP), and electric conductivity (EC), using a portable multi-parameter apparatus (HQ40d Portable Multi-Parameter Meter, Hach Lange GmbH, Germany), and chlorophyll content, using a fluorescence detector (HHLD Fluorescence-Chlorophyll, Turner Designs, USA); ii) in laboratory, in outflow of tank of fish: anion (Cl^-^, NO_3_^-^, PO_4_^3-^, SO_4_^2-^) and cation (NH_4_^+^, Na^+^, Mg^2+^, K^+^, Ca^2+^) content in the water by ion chromatography (IC) as described by Nicoletto et al. [13]. The IC was performed using an ICS-900 system (Dionex Corp., Milan, Italy) equipped with a dual piston pump, model AS-DV autosampler, isocratic column at room temperature (25°C), DS5 conductivity detector and AMMS 300 suppressor (4 mm) for anions and CMMS 300 suppressor (4 mm) for cations. Chromeleon 6.5 Chromatography Management software was used for system control and data processing. A Dionex IonPac AS23 analytical column (4 mm × 250 mm) and guard column (4 mm × 50 mm) were used for the separation of anions, whereas a Dionex IonPac CS12A analytical column (4 mm × 250 mm) and guard column (4 mm × 50 mm) were used for the separation of cations. The eluent consisted of 4.5 mmol L^-1^ sodium carbonate and 0.8 mmol L^-1^ sodium bicarbonate at a flow rate of 1 mL min^-1^ for anions and of 20 mmol L^-1^ methanesulfonic acid for cations at the same flow rate. Anions and cations were quantified following a calibration method. Dionex solutions containing four anions and five cations at different concentrations were taken as standards and the calibration curves were generated with concentrations of the standards ranging from 0.4 to 20 mg L^-1^ for the anions and from 0.5 to 50 mg L^-1^ for the cation.

### 2.3 Monitoring and feeding of fish

Health status and mortality of fish were monitored daily. All fish from each unit were weighed individually every month (five times) during the experimental period: June 27 (beginning of the cycle of catalogna), July 25 (end of the cycle of catalogna), August 29 (end of the cycle of lettuce), September 29 (half of the cycle of Swiss Chard), and November 7 (end of the cycle of Swiss Chard). The quantity of feed to be administered daily was calculated for each AP unit on the basis of the biomass present at the moment of each weighing, i.e., 2% of biomass from the beginning of the experiment to September 18, 1% from September 19 to October 11, and 0.5% from October 12 to the end of the experiment, according to the temperature of water. Fish were not fed two days before and one day after weighing.

The following variables were determined for each cycle of chicory, lettuce, and Swiss Chard and the entire experimental period:

$$Specific\;growth\;rate\;(SGR\;\%\;{day}^{-1})\;=\;\frac{\ln\;(final\;weight\;of\;fish)\;-\ln\;(initial\;weight\;of\;fish)\;}{\;days\;on\;trial}\times \;100$$ (1)

$$Feed\;conversion\;ratio\;(FCR)\;=\;\frac{total\;weight\;of\;\;\;(g)}{total\;weight\;gain\;of\;fish\;(g)}$$ (2)

### 2.4 Monitoring of plants

For the three vegetal species, two times per week and in 18 plants per unit, the content of chlorophyll (SPAD index) was estimated using a portable chlorophyll-meter (SPAD-502, Minolta, Japan). At harvesting time all plants were harvested, divided into aboveground and belowground, and fresh weight was immediately measured. After this, aboveground and belowground biomass of half the plants were dried in a thermo-ventilated oven at 65 °C until constant weight was reached to determine dry weight and dry matter content.

### 2.5 Ethics statement

The study was submitted (project no. 6/2017) to the Ethical Committee for Animal Experimentation (OPBA, Organismo per la Protezionedel Benessere Animale) of the University of Padova and approved (prot. n. 15132). All animals were handled according to the principles stated by the EC Directive 86/609/EEC regarding the protection of animals used for experimental and other scientific purposes. Abnormal behavior (i.e., erratic swimming), weight loss between two recordings, and apparent parasitic infections were used as specific endpoint criteria. Once one of the endpoint criteria was noted, the animals had to be euthanized immediately. In our trial, a total of 64 fish were used; no animal was euthanized whereas two animals died before meeting criteria for euthanasia. In the dead animals a general inflammation of the digestive apparatus was detected, but it was not associated with a specific disease.

The conditions (water temperature, dissolved oxygen, ammonia and nitrate concentrations) were constantly monitored in order to guarantee adequate environmental conditions for fish. The weight of fish was recorded under veterinarian control while maintaining the animals out of water for the minimum possible time in order to minimize the stress. Research staff involved in animal handling were animal specialists (PhD or MS in animal science).

### 2.6 Statistical analysis

The normality of data was checked using the Kolmogorov-Smirnov test. As the chemical and physical parameters of the water were not normally distributed, the Kruskal-Wallis non-parametric test at p < 0.05 was used to check the significance of differences among median values of species (catalogna, lettuce, and Swiss Chard) and treatments (APL, APH, and HP). The growth of fish, feed conversion, total biomass of fish, daily evapotranspiration of water from the systems, SPAD values, and yields of vegetables were normally distributed, and so, the analysis of variance (ANOVA) was used and when p < 0.05 the means were separated using the Tukey HSD test among species (catalogna, lettuce, and Swiss Chard) and treatments (APL, APH, and HP).

## 3. Results

### 3.1 Water

Daily consumption of water in the AP units due to evapotranspiration was not significantly different among treatments (p > 0.05), with an average value of 8.2 L d^-1^, which was equal to 1.37% of the total water of the system,. A significant effect was found according to the cycle of crops with the highest average consumption recorded during the summer cycles (catalogna 11.8 L d^-1^ and lettuce 12.3 L d^-1^) and the lowest consumptions monitored during the autumnal cycles with Swiss Chard (4.9 L d^-1^).

**Fig 2:**
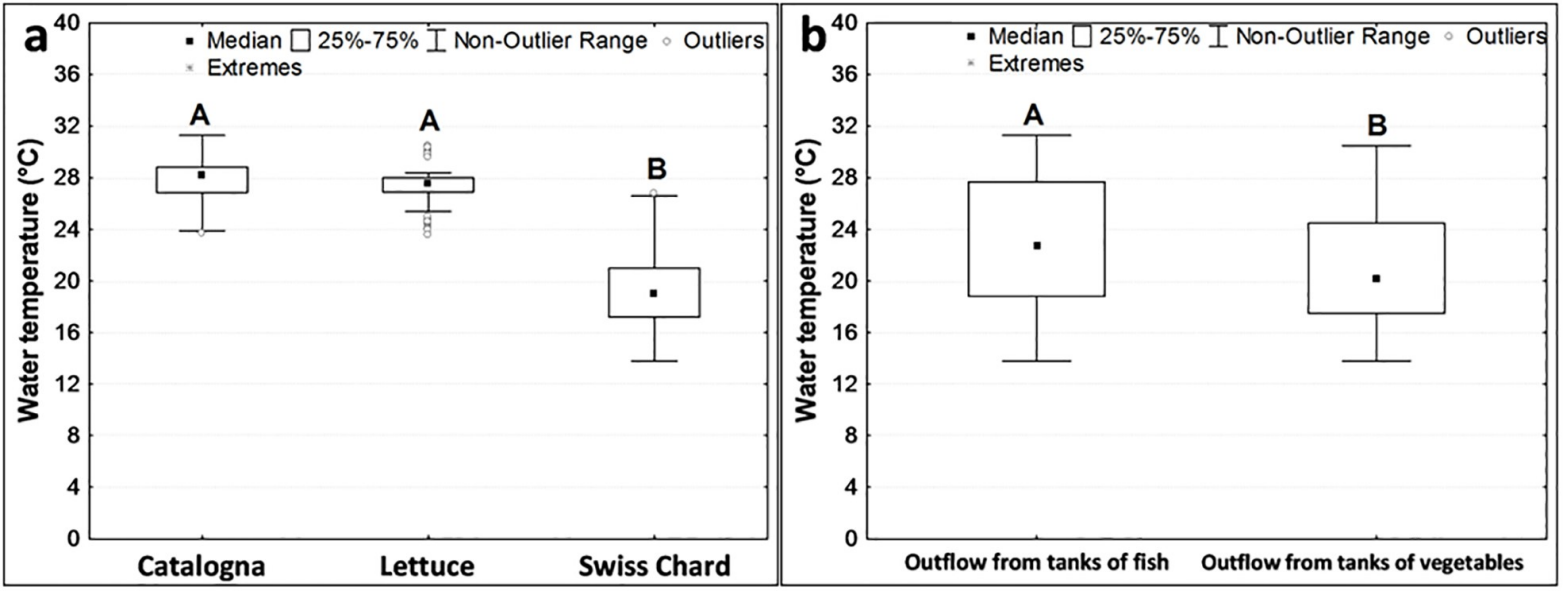
Temperature of water (a) during different cycles of crops (from June 27 to July 25: catalogna, from July 26 to August 29: lettuce, from August 29 to November 7: Swiss Chard), and (b) at different sampling points inside the systems. Different letters indicate significant differences with Kruskal-Wallis (a) and Mann-Whitney (b) tests at p < 0.05 level.

The median value for water temperature was 21.2 °C. Although this was not influenced by the treatments, significantly lower values were observed during the cycle of Swiss Chard (summer-autumn crop) than the cycles of catalogna and lettuce (summer crops) (Fig 2a). Taking into account the sampling position, the temperature of the outflow from the tanks of fish was significantly higher than the outflow from the tanks of vegetables (Fig 2b).

**Fig 3:**
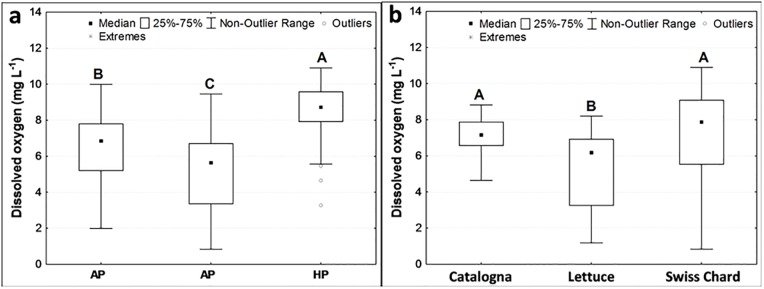
Content of dissolved oxygen in the water in the different AP treatments (a), and during different cycles of crops (b). Different letters indicate significant differences with the Kruskal-Wallis test at p < 0.05 level.

Dissolved oxygen was significantly different among treatments (Fig 3a), crops (Fig 3b), and sampling points, with a median value of dissolved oxygen of 7.8 mg L^-1^ in the outflow from the tanks with fish and 5.5 mg L^-1^ in the outflow from the tanks of vegetables.

The EC value was significantly influenced by the treatments (p < 0.05), with a median value of 1.35 dS m^-1^ in APL, 1.39 dS m^-1^ in APH, and 1.68 dS m^-1^ in HP. Considering that the EC value did not significantly change according to sampling point, the contents of anions and cations in the water are reported and discussed taking into account AP treatments and crops.

**Fig 4:**
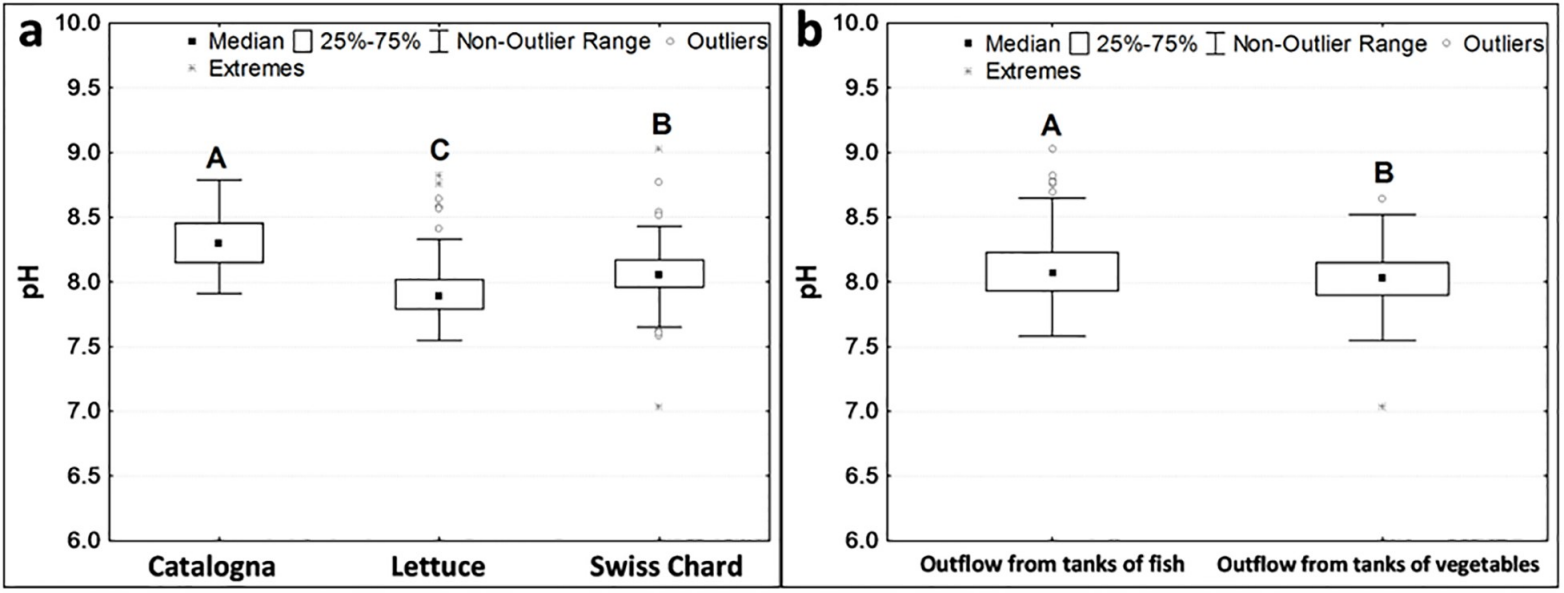
pH of water during different cycles (a) and at different sampling points (b). Different letters indicate significant differences with Kruskal-Wallis test (for a) and Mann-Whitney test (for b) at p < 0.05 level.

The pH of water ranged between 7 and 9, showing no differences between treatments. However, it was significantly different among crops (Fig 4a) and was significantly lower in the outflow from the tanks of vegetables than in the outflow from the tanks of fish (Fig 4b).

The redox potential of water ranged from -214 mV to +222 mV. The APH treatment showed a significantly lower median value (-18.7%) than the other two treatments (+132 mV). The redox potential was significantly higher during the cycle of Swiss Chard (+129 mV) than those of catalogna and lettuce (+125 mV) and it was significantly higher in the outflow from the tanks of fish (+133 mV) than the outflow from the tanks of vegetables (+115 mV).

The median value for chlorophyll in the water was significantly higher in the AP treatments than in the HP control (47 μg L^-1^ vs. 12 μg L^-1^). It was significantly lower in the outflow from the tanks of vegetables (29 μg L^-1^) than the outflow from the tanks of fish (35 μg L^-1^). Lastly, significantly higher dissolved chlorophyll (4.2 times) was observed during the cycles of lettuce and Swiss Chard than the cycle of catalogna.

Anions and cations in water were significantly different between treatments within each cycle of crops (Table 1). The highest NH_4_^+^ concentration was measured in the APH treatment and during the cycle of lettuce whereas the highest NO_2_^-^ concentrations were found during the cycle of catalogna (Table 1). Particularly, the highest NO_2_^-^ concentrations were noted during the first month of the experiment (average value 16.7 mg L^-1^) whereas after this period the NO_2_^-^ concentrations were always below 0.8 mg L^-1^ and many times below detectable threshold. The highest NO_3_^-^ concentration was detected in the HP control whereas significantly lower values were observed with increasing stocking density. The density of fish did not significantly influence the PO_4_^3-^ concentration in the water. Nevertheless, a significant increase was noted for K^+^ concentration with increasing density. An opposite trend was found for Mg^2+^ and Ca^2+^ between APL and APH treatments (Table 1). Irrespective of treatments, PO_4_^3-^ concentration in the water was higher in the second cycle (median value 74.0 mg L^-1^) than in the first (median value 48.7 mg L^-1^) and the third (median value 50.7 mg L^-1^) ones, whereas a significant median value reduction (-47%) of K^+^ was found along the three cycles of crops.

**Table 1:** Median concentrations of anions and cations in the water under different treatments and during different cycles of crops. Different letters in a row indicate significant differences between treatments during each cycle of crop using the Kruskal-Wallis test at p < 0.05 level. ns = not significant.

Parameters (mg L^-1^)	Catalogna			Lettuce			Swiss Chard		
	HP	APL	APH	HP	APL	APH	HP	APL	APH
NH_4_^+^	0.53^b^	0.57^b^	0.87^a^	0.53^c^	0.91^b^	1.63^a^	0.50^c^	0.94^b^	1.18^a^
NO_2_^-^	17.77^ns^	17.02^ns^	14.20^ns^	0.03^b^	0.05^b^	0.19^a^	0.06^b^	0.09^b^	0.17^a^
NO_3_^-^	705.0^a^	34.3^b^	3.1^c^	575.6^a^	113.4^b^	4.8^c^	406.2^a^	139.1^b^	6.5^c^
PO_4_^3-^	8.2^b^	62.9^a^	64.7^a^	17.6^b^	99.4^a^	95.3^a^	5.0^b^	53.9^a^	62.3^a^
K^+^	155.3^ns^	156.7^ns^	160.2^ns^	108.4^b^	121.0^b^	142.6^a^	64.7^c^	82.9^b^	125.5^a^
Mg^2+^	72.9^b^	80.2^a^	81.1^a^	80.0^c^	83.5^b^	87.9^a^	82.2^c^	88.3^b^	93.4^a^
Ca^2+^	188.5^a^	88.2^b^	90.5^b^	164.5^a^	102.1^b^	92.1^c^	172.2^a^	115.2^b^	96.7^c^
Na^+^	11.6^b^	11.6^b^	12.7^a^	15.0^b^	15.5^b^	17.1^a^	14.5^b^	14.2^b^	17.6^a^
Cl^-^	15.1^b^	16.1^ab^	18.6^a^	18.7^c^	22.9^b^	27.2^a^	19.3^c^	26.1^b^	30.5^a^
SO_4_^2-^	400.5^b^	410.3^ab^	423.8^a^	413.0^c^	434.3^b^	466.6^a^	426.6^b^	465.1^a^	483.5^a^

### 3.2 Vegetables

Chlorophyll content (SPAD value) of vegetables was not significantly different (p > 0.05) among treatments during the cycles of catalogna and lettuce, whereas during the cycle of Swiss Chard the average SPAD value was significantly lower (-7.1%) in HP than in the AP treatments (36.7 on average).

Marketable yield of the vegetables was significantly (p < 0.05) different among treatments for all crops (Table 2). The HP control showed the highest production only at the end of the first cycle with a higher marketable yield compared to APL (+25%) and APH (+50%). The highest production values for the second and third crops were obtained in the APL treatment, with a higher marketable yield than APH (higher by 41% for lettuce and 21% for Swiss Chard).

**Table 2:** Means (± SE) of SPAD and marketable yield (kg m^-2^) of vegetables under different treatments. Within the same crop, different letters indicate significant differences with Tukey HSD test at p < 0.05 level. ns = not significant.

Crops	SPAD values			Marketable yield (kg m^-2^)		
	HP	APL	APH	HP	APL	APH
Catalogna	58.6 ± 3.6^ns^	45.5 ± 7.7^ns^	54.1 ± 4.4^ns^	1.20 ± 0.12^a^	0.91 ± 0.14^b^	0.59 ± 0.17^c^
Lettuce	25.2 ± 1.9^ns^	17.7 ± 3.9^ns^	24.1 ± 2.4^ns^	4.05 ± 0.61^a^	3.88 ± 0.77^a^	2.34 ± 0.39^b^
Swiss Chard	42.2 ± 4.7^a^	29.3 ± 1.5^b^	35.9 ± 3.5^b^	3.87 ± 0.35^b^	5.33 ± 0.28^a^	4.17 ± 0.65^b^

### 3.3 Fish

The health of fish was very good during the whole trial and only two fish died (1 from APL and 1 from APH treatments). These two animals died without previous symptoms of disease (necroscopy showed no specific causes of death but a general inflammation of the digestive apparatus was detected). At the end of the trial, fish weighed 446 g on average, reaching a specific growth rate (SGR) of 0.74% d^-1^. The feed conversion ratio for the whole period was 1.71, and the total biomass produced was 5.66 kg m^-3^, on average.

**Fig 5:**
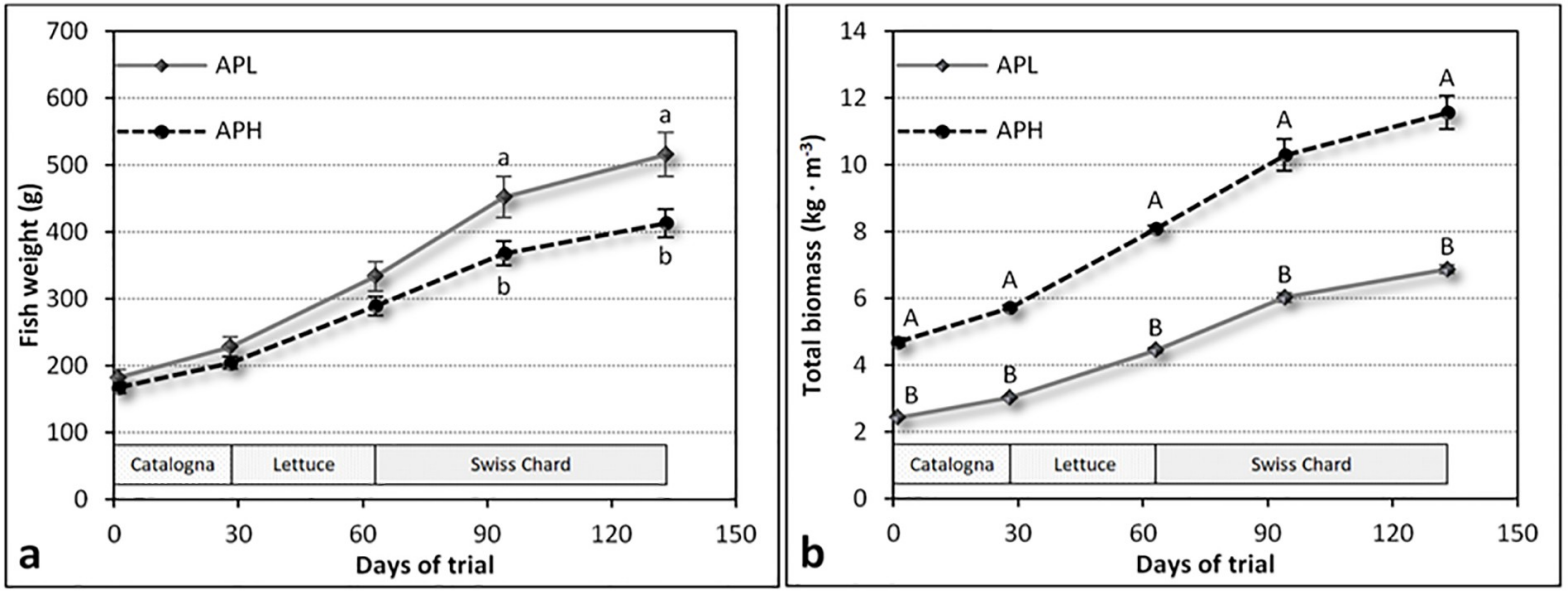
Means (± SE) of the individual body weight (a) and the total biomass (b) of fish during the different cycles according to the stocking density (APL: aquaponics with low stocking density; APH: aquaponics with high stocking density). Different letters over the graph indicate significant differences (a, b: p < 0.05; A, B: p < 0.001).

Growth performance of fish differed significantly according to the stocking density used in the AP systems. Specifically, fish of the APL treatment showed higher SGR compared to the APH (0.001 < p < 0.05) during most of the trial phases (Table 3). Also when the entire rearing period was considered, fish of the APL treatment exhibited the highest SGR (0.79% d^-1^ vs. 0.68% d^-1^ for APL and APH, respectively, p < 0.001). Accordingly, fish in the APL treatment reached a higher body weight in comparison with those of the APH treatment at about 90 day of trial in the middle of the cycle of Swiss Chard (+84 g, p = 0.05), and at the end of the study (+102 g, p = 0.01) (Fig 5a). Nevertheless, due to the higher stocking density, the total biomass of fish remained greater in the APH systems compared to the APL ones from the beginning to the end of trial (Fig 5b). Therefore, the higher the stocking density the higher the biomass of fish produced in the entire rearing period (+2.43 kg m^-3^, p = 0.01) (Table 3).

**Table 3:** Means (± SE) of growth performance and biomass of fish produced according to the stocking density in the aquaponics systems.

	Treatments		P-value
	APL	APH	
Total no. of fish/treatment	20	42	
Tanks, no.	3	3	
Average fish/tank, no.	6.7	14.0	
Specific growth rate*, % d^-1^			
0–28 days	0.82 ± 0.07	0.73 ± 0.04	0.22
28–63 days	1.08 ± 0.05	0.97 ± 0.03	0.05
63–94 days	0.98 ± 0.04	0.79 ± 0.04	< 0.01
94–133 days	0.36 ± 0.03	0.28 ± 0.02	0.02
0–133 days	0.79 ± 0.03	0.68 ± 0.01	< 0.001
Feed conversion ratio^§^			
0–28 days	2.06 ± 0.18	2.39 ± 0.22	0.31
28–63 days	1.47 ± 0.04	1.64 ± 0.10	0.19
63–94 days	1.39 ± 0.03	1.94 ± 0.20	0.24
94–133 days	1.66 ± 0.12	1.90 ± 0.11	0.21
0–133 days	1.55 ± 0.03	1.86 ± 0.12	0.07
Biomass growth^§^, kg m^-3^			
0–28 days	0.61 ± 0.05	1.03 ± 0.10	0.02
28–63 days	1.41 ± 0.05	2.38 ± 0.10	< 0.01
63–94 days	1.58 ± 0.06	2.59 ± 0.21	0.01
94–133 days	0.85 ± 0.04	1.26 ± 0.04	0.03
0–133 days	4.45 ± 0.14	6.88 ± 0.27	0.01

* Individual data,

^§^ Tank data.

No significant difference was found in terms of feed efficiency between APL and APH systems; nevertheless, in the whole trial, feed conversion tended to be more favorable in the APL treatment (-0.31 units; p = 0.07).

## 4. Discussion

### 4.1 Water

Daily rate of evapotranspiration in the experimental units was in the range reported in the literature (0.05%–5%) [3]. The values of daily evapotranspiration monitored during the cycle of lettuce (2%) were about two times higher than the values reported by Lennard and Leonard [12]. The different evapotranspiration values can be explained by: i) different average temperature of water (22 °C in Lennard and Leonard [12] vs. 27.7 °C in this study), and mainly, ii) different ratio between hydroponic surface and volume of tanks for fish which was 83% higher in this study than in that conducted by Lennard and Leonard [12].

The concentration of dissolved oxygen observed in the water was lower in APH systems than in APL ones due to: i) a higher ratio between the weight of fish and the volume of the tank, and ii) a higher organic matter accumulation in the tanks of vegetables due to feed residues and feces of fish, which may have increased the microbial consumption of oxygen for their oxidation.

Dissolved oxygen was always above the lethal threshold for European Carp (0.5 mg L^-1^) [14], but to obtain an efficient nitrification, it should be above 2 mg L^-1^ [15]. This last condition was always observed in the outflow from the tanks of vegetables in APL units showing that in this treatment, the biofilter had optimal conditions for nitrification. Conversely, in the APH system, the content of dissolved oxygen was sometimes lower than 2 mg L^-1^, indicating that the conditions for nitrification were not always optimal, as demonstrated by the NO_3_^-^ concentration (near zero) in the APH water.

The pH values of the water during the trial were in an optimal range (7.5 to 8.5). Tyson et al. [16] have reported that a pH value of 8.5 is better for *Nitrobacter* than a pH of 7.5 or lower, which is in line with Villaverde et al. [17], who reported an increase of nitrificationby +13% for each pH unit increase. In view of this, our systems had optimal pH to maximize nitrification. However this was somewhat higher than optimal values (6.5 < pH < 7.5) when taking all units of the AP system into consideration [11]. Although the high pH values of water could have reduced nutrient availability for plants, we did not observe shortages during the cycles of crops probably due to the several interactions that characterize a plant’s rhizosphere.

The ORP evolution, being indirectly related to the oxidation conditions, followed the same trend as the dissolved oxygen. Negative values of ORP were only observed in the APH system, suggesting favorable conditions for the denitrification process [18].

Considering the presence of nitrogen in the water, APH units showed the worst conditions with high values of NH_4_^+^ and NO_2_^-^, which however did not exert a negative effect on the health of fish suggesting a good buffer capacity of the AP system. The PO_4_^3-^ content in the water (near zero) in the HP treatment was due to the higher Ca^2+^ content and pH values when compared to the APL and APH treatments, whereas the increase in K^+^ content when the stocking density of fish increased, was due to the higher input of feed.

### 4.2 Vegetables

The water characteristics, together with the availability of nutrients, affected the coloring of the vegetables and the final yield. The lower SPAD values recorded for the Swiss Chard in HP are linked to the lower availability of nutrients (particularly N) when compared to the AP systems. This result agrees with the findings of Pantanella et al. [19] where the HP control showed lower SPAD values than the AP system with several crops. Moreover, our results showed that the yield in the HP was higher than in either of the AP treatments only at the end of the first cycle of crop. Thereafter, the yield in the HP was comparable to (lettuce) or lower (Swiss Chard) than that observed in the APL treatment.

The higher HP yield in the first cycle of crop was probably related to the incomplete maturation of the biofilter in the AP systems. This aspect, as reported by several authors [3, 20, 21], reduced the effective nitrification of the ammonium produced by fish, thus slowing the N availability for the plant. During the experiment, after the full activation of the biofilter, the yield of crop, especially in the APL, was comparable or higher than the control due to the continuous supply of N compounds from the fish and probably also due to the accumulation of several humic-like and protein-like dissolved organic matter components in the water [22] that exert a biostimulant effect [23, 24]. Considering the yield values, the production of catalogna was lower (-60%) than that obtained in open-field conditions [25]; the production of lettuce, on the other hand, especially for HP and APL, is in line with that reported in other soilless experiments [26]. Furthermore, the different yields observed for lettuce between HP, APL, and APH were also found by Pantanella et al. [19]. Finally, the yield of Swiss Chard in the APL was comparable to that observed by Alderman [27] in an AP system and higher (+26.4%) than that obtained by Smith et al. [28] in a pot experiment, thus confirming the high productive potential of APL.

### 4.3 Fish

Fish raised in AP can achieve growth rates comparable to those obtained with recirculating systems of aquaculture [29] and FCR within the range of conventional aquaculture [30]. Differences in species, design of AP systems, water temperature and quality, initial sizes and stocking densities of fish, composition of feed and feeding rates justify different results among studies.

In AP systems based on a low-tech closed ebb-flow substrate, Palm et al. [31] reported SGR of 0.71% d^-1^ in Nile Tilapia *Oreochromis niloticus* (174 g initial weight; 5.62 kg m^-3^ initial stocking density) and 0.65% d^-1^ in African Catfish *Clarias gariepinus* (initial weight 480 g; initial stocking density 6.72 kg m^-3^), which are nearly similar to those observed in our trial (0.74% d^-1^ on average in AP systems) in the case of tilapia, but are slightly lower in the case of African Catfish. Nevertheless, both species used in the above-mentioned study exhibited lower FCR (1.02 on average) compared to our values (1.71 on average). Differing from this, juveniles of Koi Carp *Cyprinus carpio* (initial weight 5.96 g) reared in AP at 1.4 kg m^-3^ of initial stocking density for a 45-d trial showed SGR similar to those observed in our study (0.82% d^-1^ on average), but dissimilar FCR (2.31 on average) [32].

In our conditions, the growth of fish and feed efficiency decreased mainly during the last 39 d of trial due to a reduction in water temperature (< 20 °C) and feeding rate. In conventional aquaculture systems, according to Oyugi et al. [33], European Carp juveniles reached the highest growth rate at 20 °C to 24 °C, whereas Somerville et al. [11] reported that the optimal temperature of water for European Carp is between 25 °C and 30 °C.

In our study, the SGR (-14%) and FCR (+20%) were impaired when initial stocking density was increased. The decline observed in the growth of fish in APH treatment was associated with a poor quality of water, especially in terms of higher ammonium concentrations (+50%) and lower DO levels (-12%) compared to the APL system, which reached critical values in some cases. Biswas et al. [34] reported that these characteristics of water significantly affected the health and the growth rate of European Carp. Indeed, DO levels and ammonia concentrations of water, together with other properties such as carbon dioxide, nitrate, nitrite, and pH values, represent key factors for the growth and welfare of fish in AP [35] as well as in conventional aquaculture [36, 37]. In contrast, some authors have observed reduced growth rates at increasing density regardless of the water quality, thus suggesting that physiological stress as a consequence of crowding and competition for feed and living space could inhibit the growth of fish [38, 39].

In accordance with our findings, Hussain et al. [40] have reported that the SGR of Koi Carp (*Cyprinus carpio*) (initial weight of 4.24 g) cultured in AP was strongly reduced with increasing density of fish from 1.4 kg m^-3^ to 2.1 kg m^-3^ to 2.8 kg m^-3^. Other authors observed a significant reduction of SGR with increasing stocking density in juveniles of tilapia farmed in AP [41].

Several studies have found a negative effect of increasing stocking density on feed efficiency of farmed fish in AP or conventional aquaculture [37, 40, 42, 43]. However, other authors did not observe significant changes in FCR with changes in the stocking density [38, 44, 45].

Notwithstanding the depression of growth in fish of the APH treatment, the highest stocking density resulted in a significantly higher total net yield of biomass (+55%), which has also been observed in the African Catfish cultured in cages suspended in a dugout pond by Hengsawat et al. [46].

In our study, the mortality was very low (3% on average), which suggests the possibility of maintaining good health for fish in the tested AP system, as also in the case of final stocking densities higher than 10 kg m^-3^. In the study of Knaus and Palm [47], the mortality rate of European Carp reared at 9.2 kg m^-3^ of initial stocking density reached 7.84%. In other AP systems, the mortality of Koi Carp increased (from 0% to 2%) when the density of fish increased from 2.1 kg m^-3^ to 2.8 kg m^-3^ [40]. Similar results were found by Rayhan et al. [41], who observed a significant increase in the mortality (from 0% to 7.2%) of juveniles of tilapia with increasing stocking density from 0.52 kg m^-3^ to 1.58 kg m^-3^. However, considering the available information for fish cultured in different systems, a direct relationship between stocking density and mortality was not fully demonstrated [37, 44, 46].

## 5. Conclusions

The stocking density of European Carp influenced the yield of the tested AP system with better results, in terms of water quality and production of vegetables, achieved with an initial stocking density of 2.5 kg m^-3^. Moreover, growth and feed conversion of fish were negatively influenced by stocking density, but total biomass yield increased with increasing density of fish. Considering the species-specific response of fish to different stocking densities, our findings are reliable for European Carp, and further investigations are needed to establish the more suitable stocking densities for other species raised in AP. Good production of vegetables, performance and optimum health of fish suggest that the proposed low-tech system could be successfully implemented in the field on a larger scale at low construction costs.

## Supporting information

S1 Table PLOS ONE Humane Endpoints Checklist. (PDF)
