# Supplementary material for: Effect of stocking density of fish on water quality and growth performance of European Carp and leafy vegetables in a low-tech aquaponic system
Source: PLoS One. 2019 May 30;14(5):e0217561. doi: 10.1371/journal.pone.0217561 (PMC6542511; doi:10.1371/journal.pone.0217561)
Supplement: S1 Table — (PDF) [file pone.0217561.s001.pdf]

PLOS | ONE

PLOS ONE Humane Endpoints Checklist

PLOS ONE manuscript number: **PONE-D-18-30615**

Complete the following if your study design includes death of a regulated animal as a likely outcome or planned experimental endpoint. Please also include all information in the Methods section of your manuscript.

ITEM 1. Describe whether humane endpoints* were used for all animals involved in the study.

	Recommendation	Section/Paragraph
If humane endpoints were used, report the following:*		
1	The specific criteria used to determine when animals should be euthanized	Abnormal behavior as erratic swimming; weight loss between two weight recordings; apparent parasitic infection
2	Once animals reached endpoint criteria, the amount of time elapsed before euthanasia	Immediately
3	Whether any animals died before meeting criteria for euthanasia	Two animals
If humane endpoints were **not** used, report the following:*		
1	A scientific and ethical justification for the study design, including the reasons why humane endpoints could not be used, and discussion of alternatives that were considered but could not be used	
2	Whether the institutional animal ethics committee specifically reviewed and approved the anticipated mortality in the study design	

ITEM 2. Include the following details of the study design and outcomes.

	Recommendation	Section/Paragraph
1	The duration of the experiment	133 days
2	The numbers of animals used, euthanized, and found dead (if any); the cause of death for all animals	64 animals were used. No animals were euthanized. Two animals died without previous symptoms of disease (at necroscopy no specific causes of death were found; a general

		inflammation of the digestiv apparatus was detected).
3	How frequently animal health and behavior were monitored	Daily
4	All animal welfare considerations taken, including efforts to minimize suffering and distress, use of analgesics or anaesthetics, or special housing conditions	The housing conditions (water temperature, dissolved oxygen concentration, ammonia anc nitrate concentrations) were constantly monitored in order to guarantee the best environmental conditions. Weight recordings were performed under veterinary control and maintaining the animals in aerated water for the minimum time in order t minimize the stress.
5	Any special training in animal care or handling provided for research staff	The research staff involved in animal handlings was composed by animal specialists (PhD or MS in animal science). The coordinator of the staff (Prof Gerolamo Xiccato) is a member of the Ethical Committee for Animal experiments of the University of Padova.

***Definition of a humane endpoint**

A humane endpoint is an experimental endpoint at which animals are euthanized when they display early markers associated with death or poor prognosis of quality of life, or specific signs of severe suffering or distress. Humane endpoints are used as an alternative to allowing such conditions to continue or progress to death following the experimental intervention ("death as an endpoint"), or only euthanizing animals at the end of an experiment. Before a study begins, researchers define the practical observations or measurements that will be used during the study to recognize a humane endpoint, based on anticipated clinical, physiological, and behavioral signs. These may include, for instance, body temperature or weight changes, tumor size or appearance, abnormal behaviors, pathological changes, ruffled fur, reduced mobility, body posture, or expression of specific body fluid markers. Please see the NC3Rs guidelines for more information.

ARRIVE Guidelines

PLOS ONE encourages authors to follow the Animal Research: Reporting of In Vivo Experiments (ARRIVE) guidelines for all submissions describing laboratory-based animal research and to upload a completed ARRIVE Guidelines Checklist to be published as supporting information.
